# Comparing the Effect of Silybin and Silybin Advanced™ on Viability and *HER2* Expression on the Human Breast Cancer SKBR3 Cell Line by no Serum Starvation

**Published:** 2015

**Authors:** Narges Mahmoodi, Nasrin Motamed, Seyed Hassan Paylakhi, Nosrat O. Mahmoodi

**Affiliations:** a *Department of Cell and Molecular Biology, Kish International Campus, University of Tehran, Kish, Iran.*; b *School of Biology, University College of Science, University of Tehran, Tehran, Iran. *; c *School of Biology, Damghan University, Damghan, Iran. *; d *Department of Chemistry, Faculty of Science, University of Guilan, Rasht, Iran.*

**Keywords:** Breast cancer, HER2, Silybin Advanced™, Real-time RT-PCR

## Abstract

The polyphenol silybin has anti-oxidant and anti-cancer properties. The poor bioavailability of some polyphenols (flavonoids, and terpenoids) can be improved by binding them to phosphatidylcholine (phytosome technology). Many studies have focused on the most common phytosome, silybin-phosphatidylcholine, particularly for its hepatoprotective effects. However, in recent years, studies have also been conducted to determine its anti-cancer effect. Considering that the serum starvation should not be used for studies that are not focused on cell cycle arrest, we studied the effect of silybin-phosphatidylcholine from Silybin Advanced™ in 1:2 ratio (one part silybin bound to two parts phosphatidylcholine) on *HER2 *gene expression on the SKBR3 breast cancer cell line which were cultured in complete medium (not serum deprivation). The results were compared with our previous study of silybin on *HER2* expression on SKBR3 cells.

An MTT test was used to determine concentrations for cell treatment, and the gene expression was defined by real-time RT-PCR.

Outcomes showed significant concentration- and time-dependent cell growth inhibitory effects of silybin, and silybin-phosphatidylcholine and *HER2* down regulation on SKBR3 cells. Silybin-phosphatidylcholine concentrations had a much larger inhibitory and *HER2 *down regulate effect on cell growth than the same silybin concentrations on SKBR3 cells.

## Introduction

Natural polyphenols are one of the effective anti-oxidants for inhibiting cancers because of their high efficiency, and few side effects (1-6). Silybin (silibinin), the principle component of milk thistle (*Silybummarianum*) has both antioxidant and anti-cancer properties (7-8). Recent studies have shown the inhibitory effect of silybin in different cancers, such as skin (9), colon (10), lung (11), prostate (12-13), and breast cancers (14). More importantly, it has been reported that silybin has no any significant effect on the growth of normal human prostate epithelial cells (15). 

Also, recent *in-vivo* studies for liver disease indicated that the binding of phosphatidylcholine to silybin (a phytosome) is much more effective than silybin alone, and Vitacost.com, *Inc*. announced that its bioavailability is 7 to 10 times more than silybin (16). Phytosomes show much better absorption profile and improved lipid solubility which enables them to cross the biological membrane, resulting better bioavailability (17).

After skin cancer, breast cancer is the most common cancer in women (18).* HER2 *(Human Epidermal Growth Factor Receptor 2) or also known as* ERBB2* or *EGFR2*, is one of the well-known oncogenes in breast cancer, and is over expressed in about 30% of breast cancers. Amplification or over expression of *HER2* has been shown to play an important role in progression of certain aggressive types of breast cancer (19-20). 

The most common chemoprevention monoclonal antibody for metastatic HER2^+^ breast cancers, Trastuzumab (Herceptin®), blocks HER2 receptors which prevents ErbB signaling (21-22). Considering that more than one mutation leads to cancer, herceptin effects only in some patients. Therefore, in some cases, blocking a receptor is not sufficient and does not silence the related cell signals (23-24).

**Figure 1:**
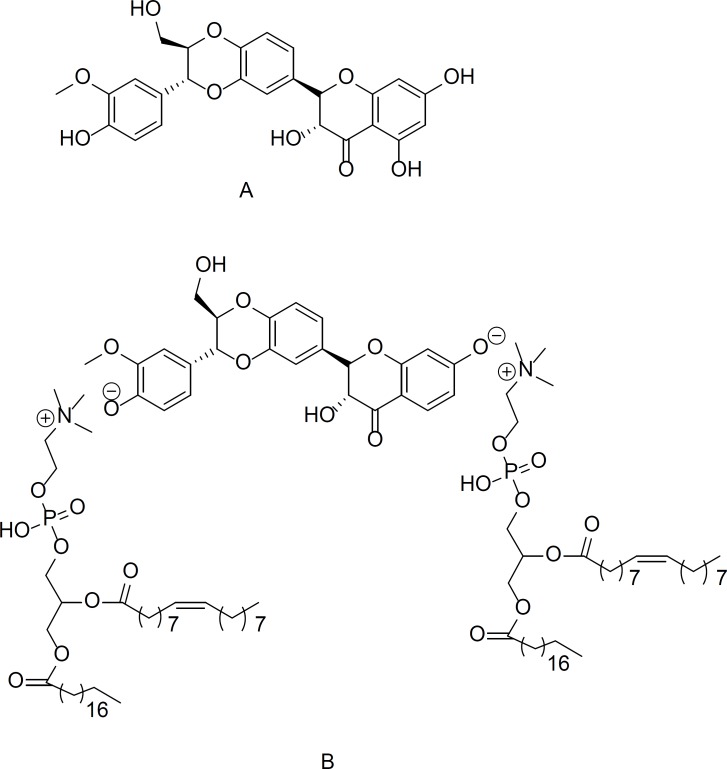
The chemical structure of A) silybin and B) silybin-phosphatidylcholine

Recent studies have suggests that finding a component that regulates gene expression would be useful for all kinds of cancer treatments. Most anti-cancer drugs for the HER2 receptor are receptor blockers, and not gene regulators. Serum starvation commonly leads to cell cycle arrest in the G0/G1 phase, and also has been used to arrest the G1 phase in cancer cells (25). In our latest research, the comparison of silybin IC_50_s, using complete medium and serum starvation procedures on BT474 or MDA-MB-453 cell line indicates that the IC_50_s reported doses by serum starvation method are less than the complete medium method (data not shown). Hence, in this study, firstly we considered that potential of silybin (natural polyphenol) (Figure 1 A) and silybin-phosphatidylcholine (we extracted from capsules) (Figure 1B) as a gene regulator for *HER2*. Secondly we evaluated if silybin-phosphatidylcholine in 1:2 ratio (not 1:1) is more effective than silybin to down regulate *HER2* gene expression on SKBR3 cell line in complete medium. 

## Experimental

*Tumor cell line and regent*

SKBR3 is naturally a human breast adenocarcinoma cell line with *HER2 *over-expression. A SKBR3 cell line was purchased from the National Cell Bank, Pasteur Institute of Iran. The cells were cultured in RPMI _1640_ medium (Invitrogen) with 15% fetal bovine serum (FBS), 1% MEM non-essential amino acids, 1% penicillin/streptomycin (all from PAA), 2 g/L sodium bicarbonate and 2.5 g/L HEPES (Sigma). SKBR3 cells were grown under standard culture conditions (37 ºC, 95% humidified air, and 5% CO_2_). For cell harvesting, 0.25% solution of trypsin (Sigma) in PBS was used.

*Separation and purification of silybin-phosphatidylcholine from a capsule*

Silybin-phosphatidylcholine was separated, and purified from Silybin Advanced™ (Enzymatic Therapy, USA). Each capsule’s ingredients were dissolved in acetone: MeOH 2:1. After filtration the solvents were subsequently evaporated to dryness. The residue was separated, purified, and characterized using IR (IR-470, Shimadzu, Japan), and ^1^H, ^13^C NMR (BrukerAvance500 MHz) spectrometers.

*Chemical treatments and MTT assay*

For the MTT assay, the cells were first seeded in three 96-well microplates. Each well contained 100 µL complete medium, and 7×10^3^ cells were seeded. The next day, the cells (with 65% density/plate area coverage) were treated with different concentrations of silybin-phosphatidylcholine (50, 75, 100, and 150 µM) for 24, 48, and 72 h. All concentrations were renewed every 24 h. The silybin-phoshphatidylcholine stock solution, 10 mM was dissolved in dimethylsulfoxide (DMSO), and MeOH at a ratio of 3:1 (26). In all tests, the final concentrations of DMSO did not exceed over 0.1% [v/v].

After the 24, 48, and 72 h treatments, the cells were incubated with 0.5 mg/mL microculture tetrazolium (Sigma) for about 3 h. The optical density (OD) of formazan dye dissolved in DMSO was measured with an ELISA microplate reader (Gen5, Power Wave XS2, BioTek, USA) at 570 nm.

The percentage of cell viability in different concentrations was calculated by the following equation:

$\mathrm{Cell viability percentage}=\frac{\mathrm{OD treated well}}{\mathrm{OD control well}}\times 100$

*IC*_50_ *determination*

Figure 3 shows the comparison of the half maximal inhibitory concentration (IC_50_) of silybin (of a previous study) (27) with silybin-phosphatidylcholine after 24, 48, and 72 h on the SKBR3 cell line. The IC_50_s was determined by probit analysis using the Pharm PCS: Pharmacologic Calculation System statistical package (Springer Verlag, USA).

After the MTT assay, and the determination of IC_50_, 75, and 100 µM silybin-phosphatidylcholine concentrations were selected which were compared with silybin 150, and 250 µM (silybin stock solution, 100 mM was dissolved in DMSO) for *HER2* gene expression evaluation after 24, 48, and 72 h. (The reason for the selection of these concentrations is explained in the last part of the Results Section).

For scrutinizing the *HER2* gene expression on SKBR3, cells were seeded in three 6-well microplates. 2 ×10^5^ cells were seeded in each well, which contained 2 mL complete medium. After 24 h, the cells were treated with 150, and 250 µM silybin, 75, and 100 µM silybin-phosphatidylcholine concentrations at 24, 48, and 72 h.

*RNA extraction and cDNA synthesis*

Total RNA was isolated from the treated cells, using an RNX Plus™ kit (CinnaGen, Tehran, Iran), CHCl_3_, IPA, and 75% EtOH (all from Atomix).The quantity and purity of the RNA samples were evaluated with 1.5% agarose gel electrophoresis with ethidium bromide, and a Nanodrop spectrophotometer (Thermo Scientific, USA).

For cDNA synthesis, 1000 ng of extracted RNA was reverse transcribed into cDNA according to the manufacturer’s protocol, using DNase I, EDTA (Cat. #PR891627), dNTP (Cat. #DN7604C, CinnaGen), and random hexamer primer (Cat.#S0142), Reverse Transcriptase 10000u (Cat. #EP0441), RiboLock RNase Inhibitor 2500u (Cat. #E00381) (all from Fermentas), DEPC Water (Cat. #MR8244C, CinnaGen).

Each RNA sample was treated with 1 µL DNase I, and 1 µL DNase I buffer, 0.5 µL RiboLock™ RNase Inhibitor, containing 10 μL reaction (30 min at 37 ºC) to remove genomic DNA contamination. 1 μL EDTA was added to deactivate DNase I for 10 min at 65 ºC. Next, 1 μL random hexamer primer (5 min at 65 ºC), 1 μL of reverse transcriptase(RT), 4 μL RT buffer, 1 μL dNTP, and 0.5 µL RiboLock RNase Inhibitor were added (Thermocycler schedule: 5 min at 25 ºC, 60 min at 42 ºC, 10 min at 70 ºC). The total reaction volume was 20 µL.

*Analysis of gene expression by real-time RT-PCR*

*HER2 *(Hs_ERBB2_1_SG QuantiTect Primer Assay, QT00060746), and *GAPDH *(Hs_GAPDH_2_SG QuantiTect Primer Assay, QT01192646) oligonucleotide primers were purchased from Qiagen, for real-time RT-PCR.

For each reaction, 1 μL cDNA was used with 1 μL related primer plus 5 µl SYBR Green I Master Mix (Quanti Fast SYBR Green PCR, Q204054) on a real-time thermo cycler (Rotor Gene 6000, Corbett Life Science, USA).The total reaction volume was 10 µL. The real-time PCR schedule was as follows: initial denaturation 95 ^o^C for 5 min, denaturation 95 ^o^C for 15 s, annealing temperature optimized from 60 to 61 ºC for 25 s, extension 72 ^o^C for 25 s. The specificity of the PCR product was assessed by verifying a single peak in the melting curve analysis.

All measurements were taken twice in duplicate, and the average was used for further analysis. GAPDH was used as a housekeeping gene, and the fold change of each target relative to GAPDH was calculated based on relative quantitation using the ΔΔC_T_ method, calculated by the 2^ –ΔΔCT^ relative expression formula.

*Statistical analysis*

**Figure 2:**
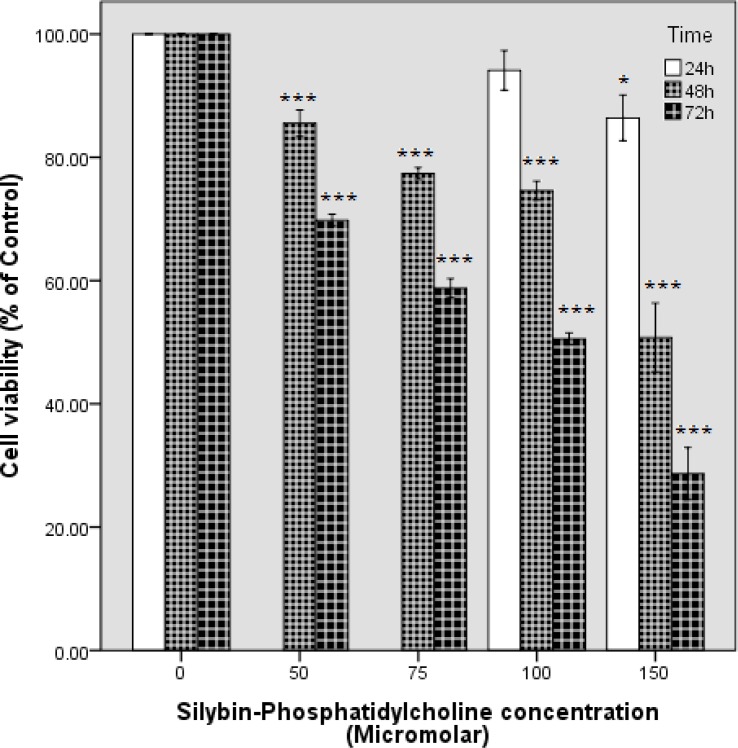
The effect of silybin-phosphatidylcholine on the cell viability of the SKBR3 breast cancer cell line. Cell viability graphs were depicted by SPSS 18 (clustered bar, summaries for group of case). Data are presented as percentage of viability in three independent experiments. Each experiment had three individual samples (Error bars: +/- 1 SD). The P values were estimated by SPSS 18, One-way ANOVA, and Dunnett-t two-sided post hoc tests

Data were analyzed using SPSS 18 software. One-way ANOVA, and Dunnett-t two-sidedpost hoc tests were employed to evaluate the statistical significance of differences between the control, and all treatments. The p-values that were considered significant are displayed *: P < 0.05, **: P < 0.01, ***: P < 0.001 in Figures 2 and 4).

## Results

*Separation and purification of silybin-phosphatidylcholine from a capsule*

Silybin-phosphatidylcholine was separated, and purified from the additives of mercantile capsule Silybin Advanced™ (supplied by Enzymatic Therapy), including cellulose, magnesium stearate, and silicon dioxide.

IR (KBr, v/cm^-1^): 3420 OH stretch, 3055 CH aromatic, 2925 CH aliphatic, 1730 CO ester, 1637 CO ketone , 1598, 1508, 1461, 1360, 1273 ether, 1210, 1179, 1082, 992, 940, 821, 722, 543, 418.

^13^C NMR (125 MHz, DMSO: CDCl_3_; 1:1, ppm): δ 14.23, 14.47, 22.89, 23.03, 25.96, 27.52, 27.55, 29.49, 29.54, 29.66, 30.00, 30.05, 31.83, 32.25, 40.17, 40.34, 54.67, 56.38, 61.53, 76.58, 78.80, 83.25, 96.34, 97.44, 100.63, 110.81, 115.60, 120.98, 128.15, 128.22, 128.39, 130.31, 130.54, 144.11, 144.41, 147.91, 163.17, 164.09, 168.36, 173.53, 173.85, 196.41.^1^H NMR (500 MHz, DMSO: CDCl_3_; 1:1, ppm): δ 0.89-0.92 (m, 12H Me), 1.28-1.58 (m, 104H CH_2_), 2.06-2.07 (m, 8H H_2_CCO), 2.29-3.07 (m, 22H Me NCH_2_), 3.49 (s, 3H OCH_3_), 3.50-3.78 (m, 6H OCH_2_), 3.78-3.96 9m, 8H OCH_2_), 5.35-5.40 (m, 6H OCH), 6.90-7,21 (m, 8H aromatic), 11.3 (s, 1H OH).

*The cytotoxicity effects of silybin, and silybin-phosphatidylcholine on SKBR3 cell line*

The cytotoxicity effects of silybin-phosphatidylcholine in four concentrations (50, 75, 100,150 µM) for 48 h, and 72 h were evaluated by the MTT assay on the SKBR3 cell line (Figure 2) and were compared with silybin in eight concentrations (50, 75, 100,150,200,250,300, 350 µM) in Table 1. Briefly, 7×10^3^ cells were seeded in 96 well plates for 24 h, and treated with different concentrations in a complete medium (no serum starvation). Cell viability graphs were depicted by SPSS 18 (clustered bar, summaries for group of case). Silybin-phosphatidylcholine treatments resulted in a concentration- and time-dependent decrease in cell viability after the 48, and 72 h treatments. However, 50 and 75 µM silybin-phosphatidylcholine were not effective in the first 24 h on SKBR3 cells cytotoxicity and the data were not reproducible, therefore, the 24 h cell viability bars were ignored for 50 and 75 µM silybin-phosphatidylcholine. These results for 100 and 150 µM silybin-phosphatidylcholine were 94.11%, and 86.40%, respectively (Figure 2). 

The comparison of silybin, and silybin-phosphatidylcholine concentrations after 48 h, shows that each silybin-phosphatidylcholine concentration had a much larger inhibitory effect on cell growth than the same silybin concentrations (Table 1). The significance for all silybin-phosphatidylcholine concentrations was P < 0.001.

Table 1 also shows that after the 72 h treatment, each silybin-phosphatidylcholine concentration had a much larger inhibitory effect on cell growth than the same silybin concentrations, and this difference was more significant than the results of 48 h. All silybin-phosphatidylcholine concentrations were statistically significant (P < 0.001).

**Figure 3:**
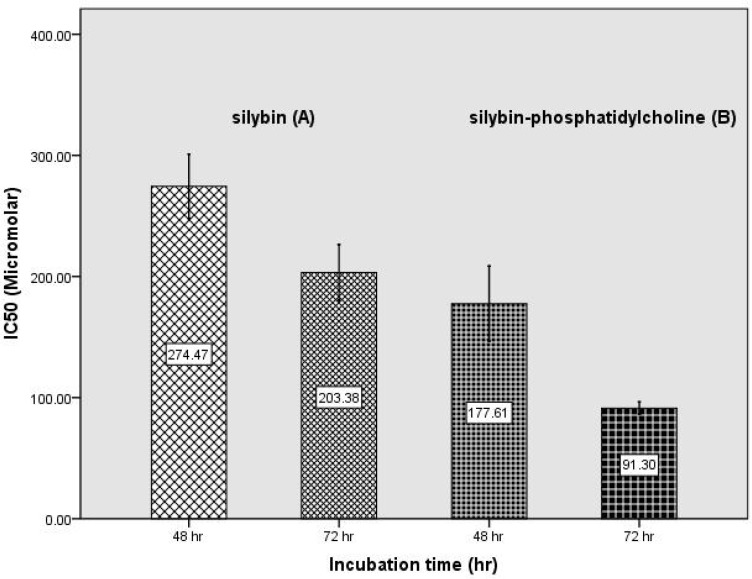
Determination of IC_50 _of silybin-phosphatidylcholine (B) during 48 h, and 72 h incubation and comparing with the IC_50_ of silybin (A) (25) (Error bars: +/- 1 SD).

Figure 3 shows the IC_50_s of silybin-phosphatidylcholine after 48, and 72 h in compared with the IC_50_s of silybin. Data from three independent experiments are presented. Each experiment had three individual samples (Error bars: +/- 1 SD). The IC_50_ comparison of silybin, and silybin-phosphatidylcholine indicated that the membrane transmission of silybin-phosphatidylcholine is 1.5 to 2.2 times more than silybin. 

**Table 1:** Comparison of silybin-phosphatidylcholine and silybin effects on the cell viability of the SKBR3 cell line

**Concentration (Micromolar)**	**Cell viability**			
	**After 48 hour**		**After 72 hour**	
	**Silybin-phosphatidylcholine**	**Silybin**	**Silybin-phosphatidylcholine**	**Silybin**
**Control**	100%	100%	100%	100%
**50**	85.53%	96.20%	69.81%	92.82%
**75**	77.38%	89.90%	58.78%	82.18%
**100**	74.61%	81.17%	50.55%	75.56%
**150**	50.77%	76.15%	28.66%	63.27%

*Down regulation of HER2 gene expression after 24, 48, and 72 h treatments with silybin, and silybin-phosphatidylcholine on SKBR3 cell line*

According to the MTT assay results, silybin-phosphatidylcholine is more effective than the same concentrations of silybin. Thus, for comparing the effect of these two compounds on *HER2* gene expression, the same concentrations were not used. Considering that the IC_50_ comparison of silybin, and silybin-phosphatidylcholine shows that silybin-phosphatidylcholine membrane transmission is 1.5 to 2.2 times greater than silybin, the silybin-phosphatidylcholine concentrations were selected half of the silybin concentrations. On the other hand, the aim of this step of the study was scrutinizing *HER2* gene expression by real-time RT-PCR (not cell mortality). Hence, all selected concentrations were less than the IC_50_s. Therefore, silybin 150, and 250 µM corresponded to 75, and 100 µM silybin-phosphatidylcholine concentrations, respectively. 

**Figure 4:**
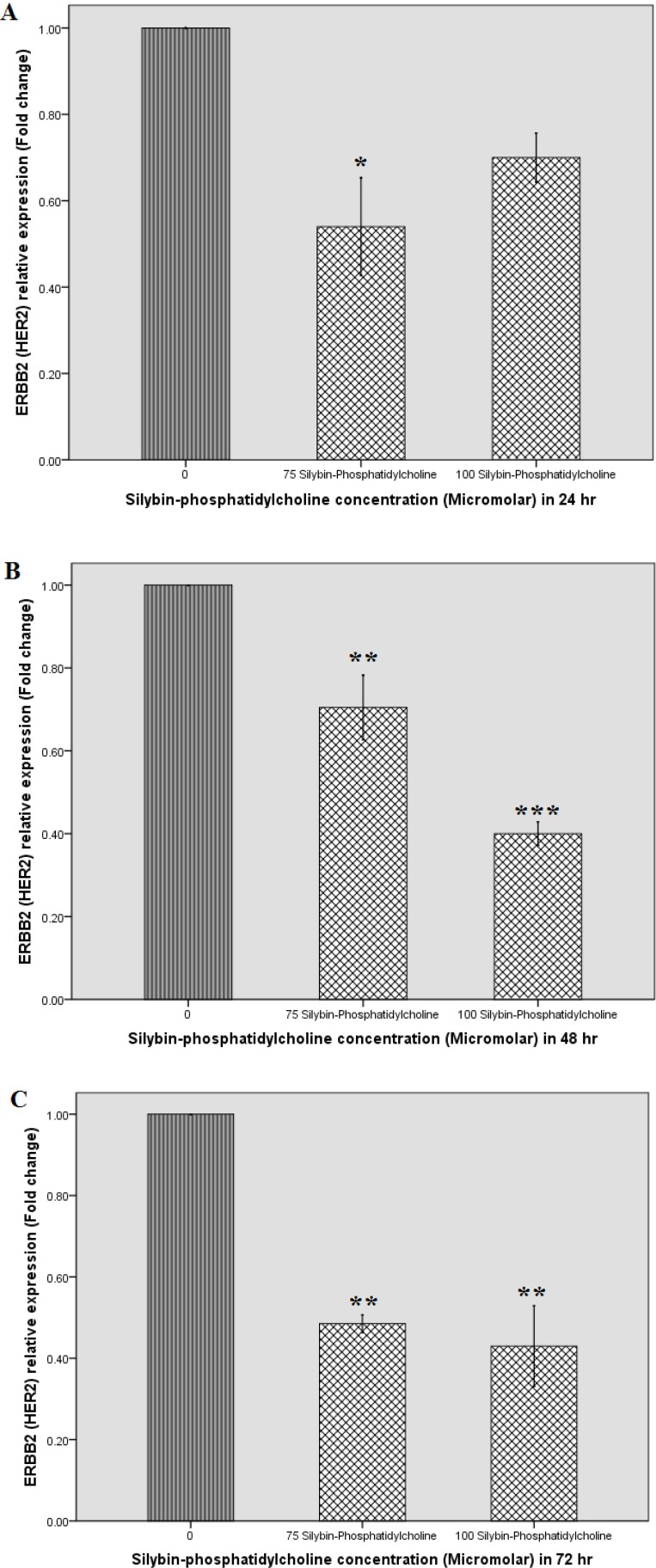
Effect of Silybin, and Silybin-phosphatidylcholine on *HER-2* mRNA expression after 24 (A), 48 (B), and 72 h (C) on the SKBR3 breast cancer cell line by real-time RT-PCR. Relative expression graphs were depicted by SPSS 18 (simple bar, summaries for group of case). Data were presented as two independent experiments. Each experiment had two individual samples (Error bars: +/- 1 SD). The p-values were estimated by SPSS 18, One-way ANOVA, and Dunnett-t two-sided post hoc tests.

Figure 4A indicates that silybin-phosphatidylcholine concentrations down regulate *HER2* gene expression after the first 24 h. As shown in Figure 4 (A, B, C), all selected concentrations, and times (24, 48, and 72 h) for silybin-phosphatidylcholine indicate *HER2* down regulation on SKBR3 cells. Table 2 indicates all concentrations (except 100 µM silybin-phosphatidylcholine) significantly down regulate *HER2* expression. In the first 24 h treatment, 150, and 250 µM silybin, and 75 µM silybin-phosphatidylcholine concentrations down regulate *HER2* mRNA expression to almost the same degree.

As shown in Table 2, the level of HER2 down regulation of 100 µM silybin-phosphatidylcholine after 48 h is more than its corresponding concentration (250 µM silybin). After 48 h, 150 µM silybin seems more effective than its corresponding concentration (75 µM silybin-phosphatidylcholine). However, the effect of 150 µM silybinon *HER2* down regulation is similar to 100 µM silybin-phosphatidylcholine (P< 0.01, P < 0.001).

After 72 h treatment, the two 75 and 100 µM silybin-phosphatidylcholine concentrations seem more effective than the corresponding concentrations of silybin, 150 µM, and 250, respectively. 

**Table 2:** Comparison of silybin-phosphatidylcholine and silybin effects on HER2 gene expression on SKBR3 cell line after 48 and 72 hours

**Time **(hour)	**HER2 Gene expression (Fold change)**					
	**Silybin concentration** (Micromolar)			**Silybin-phosphatidylcholine concentration** (Micromolar)		
	**control**	**150 **	** 250**	**control**	**75**	**100**
**After 24 h**	1	0.5	0.53	1	0.54	0.70
**After 48 h**	1	0.36	0.645	1	0.705	0.4
**After 72 h**	1	0.755	0.565	1	0.485	0.43

## Discussion

The comparison of silybin and extracted silybin-phosphatidylcholine indicates that all silybin-phosphatidylcholine concentrations had a much larger inhibitory effect on cell growth, 2 to 2.5 times more than the same silybin concentrations on SKBR3 cells. This difference was more significant according to time duration (Figure 3). Also in our previous study silybin-phosphatidylcholine was 2.5 to 3 times more effective than silybin on T47D cell lines, and showed silybin and silybin-phosphatidylcholine significantly down regulated *ESR1 *after 48 and 72 hours (26). The MTT assay results reported in this study show significant concentration and time dependent cell growth inhibitory effects (CGIE) of silybin-phosphatidylcholine on SKBR3 cells after 48 h, and 72 h. The comparison of the 24 h results, indicates that silybin and silybin-phosphatidylcholine are effective only at high conc. *e.g*. > 300 µM and > 100 µM of silybin and silybin-phosphatidylcholine respectively on SKBR3 cells. A similar result also reported in a previous study by propidium iodide staining (28). In addition, it has been reported that* HER2* over expressing tumors are resistance to various types of chemo- and endocrine therapy (29). However, in our previous studies on silybin inhibition on MDA-MB-231 breast (30), and PC-3 prostate cancer cell lines (31), all silybin elective concentrations had growth inhibitory effects in 24 h. This indicates that the SKBR3 cell line have more resistance to silybin, and silybin-phosphatidylcholine in the first 24 h than MDA-MB-231 and PC-3 cancer cell lines. A review study also reported that silybin inhibited EGFR (HER1) activation by EGF in EGFR expressing cells and involves the EGFR signaling pathway and has announced that silymarin and silybin strongly decreased cell proliferation and angiogenesis, and enhanced apoptosis in the prostate tumor xenografts, and also inhibits the EGFR pathway (32). In another study, it was reported that down-regulation of HER2 expression is not only cytostatic, but it also results in the activation of apoptotic cell death pathways in cells that overexpress HER2 (33).

We considered the effect of silybin-phosphatidylcholine on *HER2 *gene expression on SKBR3 breast cancer cells by real-time RT-PCR and compared with our previous results of the effect of silybin (27). Most concentrations showed significant *HER2* down regulation after 24, 48, and 72 h. In the first 24 h, almost all elective concentrations down regulated *HER2 *gene expression to nearly the same extent, obviously indicating that for optimum effects of silybin, and silybin-phosphatidylcholine on *HER*2 gene regulation, more than 24 h treatment is required (Table 2). The 48 h gene expression results indicated that in 150 µM silybin, and 100 µM silybin-phosphatidylcholine conc. the highest down regulation were achieved. The results for the 72 h treatment showed that 75 µM and 100 µM silybin-phosphatidylcholine concentrations seem more effective than the corresponding silybin, 150 µM and 250 µM concentrations respectively.

## Conclusion

In conclusion, both of silybin and silybin-phosphatidylcholine down regulated* HER2* expression on SKBR3 breast cancer cells. However, silybin-phosphatidylcholine is more effective than silybin for *HER2* down regulation on SKBR3 cells, and the absolute significant difference was achieved after 48 h. The outcome of *HER2* down regulation reduces cell viability, which is confirmed by the MTT results. We anticipate that less silybin-phosphatidylcholine dose could be used, by showing more effect than the same silybin dose and may leads to fewer side effects for patients.
